# Supplementary material for: Genetic and Phenotypic Parameter Estimates of Body Weight and Egg Production Traits of Tilili Chicken in Ethiopia
Source: Animals (Basel). 2025 Sep 10;15(18):2656. doi: 10.3390/ani15182656 (PMC12466866; doi:10.3390/ani15182656)
Supplement: Supplementary file 1 [file animals-15-02656-s001.zip › animals-3795832-supplementary.pdf]

Table S1. Vaccination protocol implemented at Tilili chicken selective breeding program

Age in days	Disease vaccination for	Name of vaccine	Way of Administration
0	Marek's disease virus	Mareks	Sub-cutaneous
7	Newcastle disease virus	HB1	Ocular
14 and 28	Infectious Bursal Disease	Gumboro	Ocular/water
21, 63, 112, 196, 270	Newcastle disease virus	Lasota	water
45 and 90	Fowl typhoid	Fowl typhoid	Sub-cutaneous
72	Fowl pox	Fowl pox	Wing-web

Table S2. Analysis of variance (ANOVA) for Body weight traits

Trait	Fixed effects	Sum sq	DF	F value	Pr(>F)
Hatch Weight	Generation	2355.8	1	170.3610	<2e-16 ***
	Sex	9.5	1	0.6896	0.4064
	Residuals	18903.7	1367		
Body weight at 2 weeks of age	Generation	62006	1	60.5118	1.498e-14 ***
	Sex	2423	1	2.3648	0.1244
	Residuals	1310588	1279		
Body weight at 4 weeks of age	Generation	625795	1	126.4472	<2e-16 ***
	Sex	844	1	0.1706	0.6796
	Residuals	6329853	1279		
Body weight at 6 weeks of age	Generation	907097	1	64.2090	2.474e-15 ***
	Sex	118473	1	8.3861	0.003844 **
	Residuals	18323069			
Body weight at 8 weeks of age	Generation	1969158	1	91.434	< 2.2e-16 ***
	Sex	661609	1	30.721	
	Residuals	27695703	1286		
Body weight at 10 weeks of age	Generation	5320820	1	148.101	< 2.2e-16 ***
	Sex	2927408	1	81.482	< 2.2e-16 ***
	Residuals	45663286	1271		
Body weight at 12 weeks of age	Generation	12576998	1	1271	< 2.2e-16 ***
	Sex	8501842	1	149.57	< 2.2e-16 ***
	Residuals	72247450			
Body weight at 14 weeks of age	Generation	19830284	1	299.87	< 2.2e-16 ***
	Sex	17614416	1	266.36	< 2.2e-16 ***
	Residuals	83124332	1257		
Body weight at 16 weeks of age	Generation	22538035	1	288.58	< 2.2e-16 ***
	Sex	33176714	1	424.79	< 2.2e-16 ***
	Residuals	106764047	1367		

—statistically significant at $p < 0.01$, *—statistically significant at $p < 0.001$

Table S3. Analysis of variance (ANOVA) for egg production traits

Trait	Fixed effects	Sum sq	F value	Pr(>F)
Egg production in month 1	Generation	777.3	81.977	< 2.2e-16 ***
Egg production in month 2	Generation	6003.2	517.39	< 2.2e-16 ***

Egg production in month 3	Generation	14980	828.59	< 2.2e-16 ***
Egg production in month 4	Generation	3451.7	153.28	< 2.2e-16 ***
Egg production in month 5	Generation	923.9	35.157	5.857e-09 ***
Egg production in month 6	Generation	3487.3	117.13	< 2.2e-16 ***
Cumulative egg number, month 1-2	Generation	11101	491.98	< 2.2e-16 ***
Cumulative egg number, month 1-4	Generation	82086	963.97	< 2.2e-16 ***
Cumulative egg number, month 1-6	Generation	141343	551.31	< 2.2e-16 ***
Average egg weight	Generation	714.7	37.537	1.884e-09 ***

***—statistically significant at $p < 0.001$.

Table S4. Variance components and heritability of growth traits of Tilili chicken

Trait	N	σ^2_g	σ^2_p	h^2	P-value
HW	1370	4.7171	13.92	0.33 ± 0.13**	0.005567052
BW2	1282	363.73	1056.5	0.31 ± 0.08***	0.00005331235
BW4	1282	2007.0	5120.5	0.28 ± 0.01***	0.0000000001
BW6	1300	5658.0	14706	0.29 ± 0.08***	0.0001444807
BW8	1289	10347	22578	0.25 ± 0.01***	0.00000000001
BW10	1274	12667	38052	0.31 ± 0.08***	0.00005331235
BW12	1274	22103	59627	0.29 ± 0.07***	0.00001715028
BW14	1260	23468	69470	0.31 ± 0.08***	0.00005331235
BW16	1370	27765	79835	0.34 ± 0.08***	0.00001068853

HW, BW2, BW4, BW6, BW8, BW10, BW12, BW14 and BW16, hatch weight, body weight at 2, 4, 6, 8, 10, 12, 14 and 16 weeks of age respectively; σ^2_g, σ^2_p, h^2, additive variance, phenotypic variance and heritability. **—statistically significant at $p < 0.01$, *** —statistically significant at $p < 0.001$.

Table S5. Variance components and heritability of egg production traits of Tilili chicken

Trait	Records, N	σ^2_g	σ^2_p	h^2	P-value
EPM1	473	2.935	9.733	0.30 (0.13) *	0.01050813
EPM2	473	3.041	11.875	0.26 (0.13) *	0.02275013
EPM3	473	2.612	18.266	0.14 (0.11) ^{NS}	0.1015574
EPM4	473	2.542	22.334	0.12 (0.11) ^{NS}	0.1376564
EPM5	473	7.906	26.536	0.30 (0.12) ***	0.00008841729
EPM6	473	2.526	29.901	0.08 (0.01) ***	0.00000000001
EPM12	473	8.89	23.44	0.37 (0.12) **	0.001023479
EPM14	473	13.46	84.82	0.15 (0.12) ^{NS}	0.1056498
EPM16	473	36.01	256.76	0.15 (0.11) ^{NS}	0.08634102
AEW	473	3.1506	8.61	0.36 (0.10) ***	0.0001591086

M1, M2, M3, M4, M5, M6, M12, M14, M16, egg numbers in month 1, 2, 3, 4, 5, 1 to 2, 1 to 4, and 1 to 6; AEW, Average Egg Weight. σ^2_g, σ^2_p, h^2, additive variance, phenotypic variance, environmental variance and heritability. *—statistically significant at $p < 0.05$, **—statistically significant at $p < 0.01$, ***—statistically significant at $p < 0.001$, NS— not statistically significant ($p \geq 0.05$).
